# Case Report: Paclitaxel-Induced Pneumonitis in Early Breast Cancer: A Single Institution Experience and Review

**DOI:** 10.3389/fonc.2021.701424

**Published:** 2021-06-23

**Authors:** Luke Ardolino, Brandon Lau, Isabella Wilson, Julia Chen, Linda Borella, Emily Stone, Elgene Lim

**Affiliations:** ^1^ Department of Medical Oncology, Garvan Institute of Medical Research, Darlinghurst, NSW, Australia; ^2^ St. Vincent’s Clinical School, University of New South Wales, Darlinghurst, NSW, Australia; ^3^ Department of Medical Oncology, Fiona Stanley Hospital, Murdoch, Perth, WA, Australia; ^4^ Department of Medical Oncology, Crown Princess Mary Cancer Centre, Westmead, NSW, Australia; ^5^ Department of Medical Imaging, St Vincent’s Hospital, Darlinghurst, NSW, Australia; ^6^ Department of Thoracic Medicine, St Vincent’s Hospital, Darlinghurst, NSW, Australia

**Keywords:** chemotherapy, pneumonitis and pulmonary toxicity, early breast cancer, management of toxicities, immunotherapy

## Abstract

Taxane-based chemotherapy regimens are in widespread use as standard of care treatment for patients with early breast cancer, though rarely its use can be complicated by taxane-induced pneumonitis (TIP). While breast cancer is the most diagnosed cancer in women worldwide, TIP remains under-described in this setting. Key questions relate to its incidence, diagnosis and management, potential predictive biomarkers, and the balance between this life-threatening toxicity and curatively intended treatment. At a single Australian institution, 6 cases of TIP are identified among 132 patients treated with a paclitaxel-containing regimen for early breast cancer (4.55%, 95% confidence interval 1.69-9.63%). This review first outlines the presentation, management, and outcomes for these cases, then answers these questions and proposes an approach to suspected TIP in patients with breast cancer.

## Introduction

Taxane-induced pneumonitis (TIP) usually occurs within days-weeks following treatment. It is rare and poorly characterised, with incidence cautiously estimated to be 1-5% (1), yet it is critical that it be diagnosed and managed early, as the consequences are potentially fatal. Most published cases are in non-small cell lung cancer (NSCLC) or haematological malignancies and, despite the widespread use of taxanes in breast cancer, there are only a few individual case reports and a small case series in this tumour type.

Taxanes are microtubule toxins used to treat a wide range of malignancies. Commonly used taxanes include docetaxel, cabazitaxel, and paclitaxel. The most common paclitaxel-related toxicities are hypersensitivity reactions, neuropathies and haematological toxicities, most commonly neutropenia (2). More rarely, paclitaxel can induce an interstitial pneumonitis, often resulting in significant clinical deterioration and cessation of further chemotherapy. Nanoparticle-albumin bound paclitaxel (nab-paclitaxel) theoretically carries a reduced risk of hypersensitivity reactions because it lacks many of the solvents found in conventional paclitaxel (3). Interestingly, nab-paclitaxel is associated with lower rates of pneumonitis and a milder clinical course, perhaps leading to underreporting in clinical trials.

**Figure 1:**
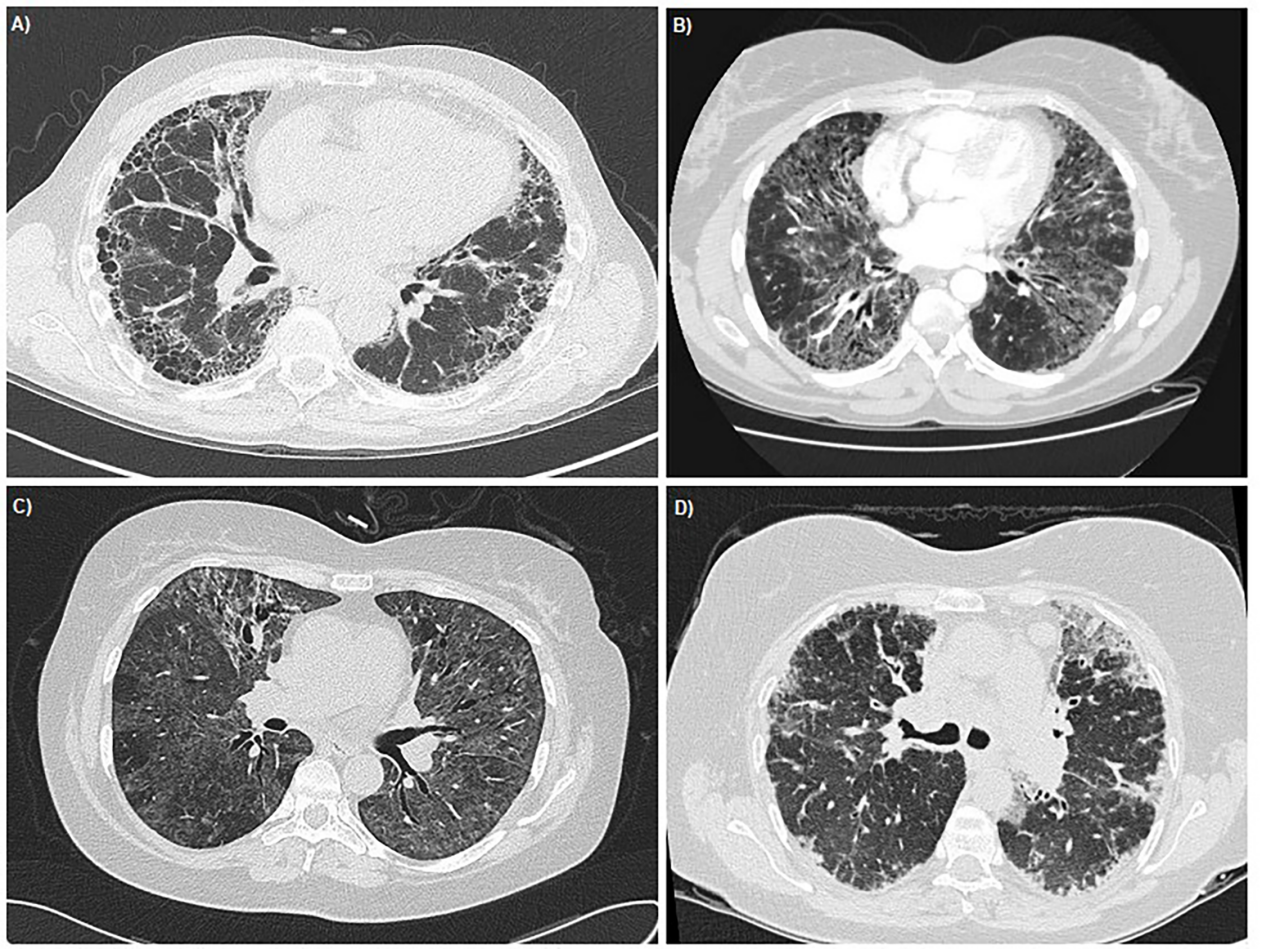
Thoracic HRCT images demonstrating the radiological appearances of the four sub-types of TIP. **(A)** Pulmonary Fibrosis - Case courtesy of Dr Ian Bickle, Radiopaedia.org, rID: 26493 **(B)** Acute Diffuse Interstitial Pneumonia - Case courtesy of Radswiki, Radiopaedia.org, rID: 11516 **(C)** Sub-acute Diffuse Interstitial Pneumonia - Case courtesy of Dr Mark Holland, Radiopaedia.org, rID: 19551 **(D)** Pulmonary Opacities with peripheral eosinophillia - Case courtesy of Dr Lawrence Josey, Radiopaedia.org, rID: 18019.

Taxane induced pneumonitis (TIP) can occur through a variety of different mechanisms and take a variety of forms. Respiratory symptoms may start at the first treatment and worsen with subsequent exposure or after several cycles. Several clinical entities have been observed: 1) acute diffuse interstitial pneumonia is the most severe and quickly progresses to acute respiratory failure; 2) subacute diffuse interstitial pneumonitis has delayed onset and a less severe clinical course; 3) pulmonary opacities with peripheral eosinophilia are exceedingly rare marked by an indolent course and excess infiltration of eosinophils within lung interstitium and alveoli. Pulmonary fibrosis can occur as a late complication following the initial inflammatory process and may result in chronic respiratory failure (4). Radiological examples of these four sub-types of TIP are presented in **Figure 1**. Mechanistically, TIP is thought to be the result of delayed hypersensitivity reaction, suggested by positive leukocyte migration inhibition test to paclitaxel in lymphocytes taken during bronchoalveolar lavage of affected patients (5). Curiously, there have even been cases of TIP following insertion of paclitaxel-containing coronary artery drug eluting stents (DES) (6).

## Predisposing Factors to TIP

There are a number of predisposing factors to TIP which should prompt a lower threshold for investigating respiratory symptoms in patients receiving taxanes. These include the following

### Pre-Existing Interstitial Lung Disease (ILD)

ILD includes a group of pulmonary disorders that result in radiological patterns that vary according to underlying histology. High resolution computed tomography (HRCT) is the most sensitive imaging examination to assist with differentiation of types of ILD. HRCT findings primarily reflect interstitial fibrosis and commonly include increased reticular markings, non-dependent ground glass opacities, traction bronchiectasis, and honeycombing. In a retrospective series of 392 Japanese patients with NSCLC treated with 3-weekly docetaxel (60 mg/m^2^), TIP was observed in 26% of those with pre-existing ILD compared to 4.6% in the overall cohort. Analyses of patients with underlying ILD treated with docetaxel demonstrated an acute exacerbation of the ILD in 14-18% of patients, with half of these exacerbations resulting in death. Resultantly, it is the view of many medical oncologists that baseline ILD is a relative contraindication to taxane chemotherapy (7).

### Use of Radiotherapy

The use of taxanes in combination with either concurrent or sequential radiotherapy (RT) also appears to increase the risk of TIP. However, sequential rather than concurrent administration of thoracic RT with taxane chemotherapy, does appear to negate some of this risk, with an overall lower incidence of pneumonitis (HR = 0.29. [p = 0.003]) (8–10). Additionally, taxane based chemotherapy (especially paclitaxel) does appear to increase the risk of radiation pneumonitis and radiation recall independently of prior TIP, although this risk remains low (11, 12).

### Combination Systemic Therapy

The incidence of TIP is higher when taxanes are combined with other cytotoxic agents (2) and especially with gemcitabine (13, 14). In one case series, 4 of 12 patients (33%) with NSCLC who were treated with the combination of paclitaxel and gemcitabine developed CTCAE ≥ grade 2 pneumonitis (15). In two further case series, ≥ grade 3 pneumonitis was observed in 4 of 39 (10%) and 7 of 63 (11%) patients treated with combination paclitaxel plus gemcitabine or docetaxel plus gemcitabine, respectively (13, 14). Finally, a meta-analysis describing TIP in 5,065 patients receiving docetaxel with gemcitabine (22.1% breast cancer) demonstrated an overall TIP incidence of 2.7% (95% CI 2.26-3.14) for ≥ grade 3 lung toxicity. The lung cancer and breast cancer specific incidence were 4% (95% CI 3.68-4.32) and 0.8% (95% CI 0.68-0.87) respectively. Relative to patients with lung cancer, patients with breast cancer developed severe lung toxicity less frequently (OR = 0.18, 95% CI (0.09, 0.36). Among cases of TIP, mortality was 0.35% in the overall population and in this setting, patients with lung cancer, compared to breast cancer, did not show significantly more fatal lung toxicity (OR = 0.20, 95% CI (0.02, 1.67) (16).

### Taxane Dose and Schedule

While observational studies have found paclitaxel doses greater than 250mg/m2 to be associated with pneumonitis risk, there was no dose-response relationship seen with lower doses (17). Certainly, the standard schedule of weekly paclitaxel (80mg/m^2^) used in breast cancer following AC is well below this dose threshold. When compared with 3 weekly paclitaxel therapy (175 mg/m^2^), weekly dosing may be associated with increased incidence and severity of pneumonitis. Two comparative trials comparing weekly with every-three-week paclitaxel in women with advanced breast cancer, demonstrated rates of grade 3 or higher dyspnoea during therapy of 5-7% versus 3-4% respectively (3, 17). Additionally, in a further phase III trial comparing weekly to three-weekly docetaxel, the overall incidence of TIP was 27% and 6% respectively (7), however, the incidence of lung toxicity of ≥ grade 3 was low in both groups (5% and 3% respectively), similar to paclitaxel. Therefore, although a weekly dosing schedule does appear to increase the incidence of TIP, it is unlikely that there is any significant difference between paclitaxel and docetaxel (18). 

## Management of TIP

When presented with an acutely breathless and hypoxic patient who has received paclitaxel, the initial assessment and treatment must consider alternative aetiologies including pulmonary embolism, lymphangitis, and infection, in particular atypical infections such as Pneumocystis Carinii which can arise in immunocompromised hosts such as patients undergoing chemotherapy or patients receiving dexamethasone as part of their treatment regimen. Supplemental oxygen and empirical antimicrobials can be provided while awaiting the results of imaging. In patients with radiological features consistent with TIP, such as organising pneumonia (OP) or non-specific interstitial pneumonia (NSIP) patterns, who also have a compatible clinical presentation, bronchoscopy should be considered and bronchoalveolar lavage (BAL) performed. Fluid should be examined for hemosiderin deposition, lipid-laden macrophages, Langerhans cells, and malignant cells. If TIP is suspected, glucocorticoid therapy should be initiated. This is based on case reports and anecdotal observational essays of patients’ respiratory failure being successfully reversed with this treatment (2, 18, 19). The putative mechanism is by suppressing the hypersensitivity reaction driving the pneumonitis (6). While oral prednisone dosed at 0.7mg/kg/day (40-60 mg) is usually adequate to treat TIP, intravenous methyl-prednisone may be required for impending respiratory failure dosed at 1-2mg/kg/day (19, 20). Following clinical recovery, the glucocorticoid should be slowly tapered over 1-2 months.

Here, we report six cases of patients with early-stage breast cancer diagnosed with TIP in the setting of dose-dense AC chemotherapy followed by weekly paclitaxel. To the authors’ knowledge, this represents the largest breast-specific case series in the literature and expands on our current knowledge from previous smaller case studies.

## Case Series

Between 1 March 2018 to 1 March 2021, 132 patients received a paclitaxel-containing chemotherapy regimen for either adjuvant or neoadjuvant treatment of early-stage breast cancer. Six patients were diagnosed with TIP. This provides a point estimate of TIP incidence at our centre of 4.55% (95% confidence interval 1.69-9.63%), similar to what has been previously described (2).

## Demographics

The median age of our cohort was 57, and all patients were prescribed dose-dense doxorubicin (60mg/m^2^) and cyclophosphamide (600mg/m^2^) followed by weekly paclitaxel (80mg/m^2^) for treatment of early-stage breast cancer. Clinicopathological features of the breast cancer diagnoses are described in **Table 1**. No patients had pre-existing ILD detectable on baseline computed tomography (CT) staging imaging and all were never-smokers. One had received prior preoperative radiotherapy for a fungating primary breast carcinoma, which was completed 17 weeks prior to developing TIP. Five patients were planned for subsequent adjuvant radiotherapy.

**Table 1:** Summary of patient’s demographics, diagnosis and management.

Case	Age	ER status	HER-2 status	Nodal status	Onset of symptoms since commencing paclitaxel	ILD on staging CT	Pattern	DLCO (mL/min/mmHg)/KCO (mL/min/mmHg/L) at time of diagnosis	Received anthracycline prior to TIP	Received Corticosteroids	Adjuvant RT following paclitaxel
1	67	Negative	Negative	Negative	16 days	Negative	NSIP	15.3 (64%)/	Yes	Yes	Yes
								4.4 (77%)			
2	44	Negative	Negative	Negative	7 days	Negative	OP	13.6 (54%)/	Yes	No	Yes
								3.5 (66%)			
3	62	Positive	Negative	Positive	2 days	Negative	OP	16.3 (74%)/	Yes	Yes	Yes
								3.5 (73%)			
4	63	Negative	Positive	Negative	15 days	Negative	OP	15.2 (66%)/	Yes	Yes	No
								4.5 (71%)			
5	37	Positive	Positive	Positive	17 days	Negative	NSIP	Not performed	Yes	No	Yes
6	52	Negative	Positive	Negative	20 days	Negative	OP	17.7 (71%)/	Yes	Yes	Yes
								3.92 (81%)			

CT, Computerised Tomography; ILD, Interstitial lung disease; NSIP, Non-specific interstitial pneumonia; OP, Organising pneumonia; RT, Radiotherapy; TIP, Taxane induced pneumonitis.

## Diagnosis of TIP

**Figure 2:**
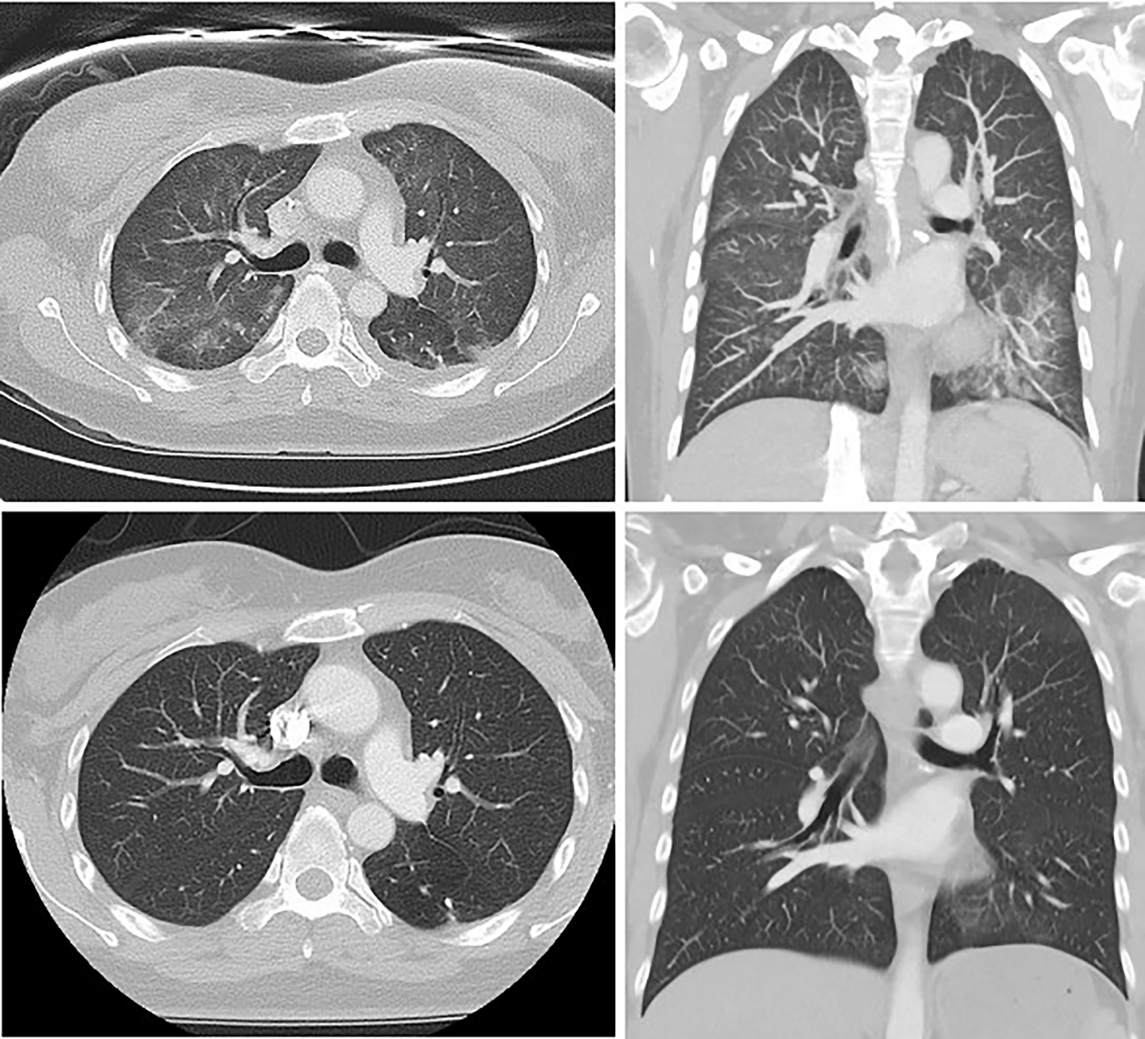
Thoracic HRCT from patient 4, demonstrating interstitial fibrosis, increased reticular markings, ground glass attenuation and traction bronchiectasis at the time of diagnosis (above) and subsequent standard-resolution thoracic CT showing resolution of these changes following 6 weeks of corticosteroids (below).

There was a median of 15 days (range 2-20) from the first paclitaxel infusion until development of pneumonitis. All patients developed a dry cough, dyspnoea, and fever (T ≥38°C). Four out of the five patients described additional significant lethargy. All patients were admitted to hospital as inpatients for respiratory support and further diagnostic work up following clinical presentation. All received supplemental oxygen and empirical intravenous antibiotics. Serum C-reactive protein was elevated in all patients, with median 105mg/L (range 26-270), though none had neutrophilia or eosinophilia. All patients had chest radiographs showing diffuse, bilateral interstitial opacities, and two patients had bilateral pleural effusions. All underwent initial CT of the chest, with four patients proceeding to HRCT: all had features of pneumonitis with specific patterns outlined in **Table 1**. A radiological example of TIP with NSIP pattern is presented in **Figure 2**.

All patients underwent pulmonary function testing (PFT), with median diffusing capacity for carbon monoxide (DLCO) of 15.2 mL/min/mmHg (66% predicted; normal 75-140% predicted). In the one case with pre-treatment PFTs available, DLCO decreased from 16.8 mL/min/mmHg (67% predicted) to 13.6 mL/min/mmHg (57% predicted). In the one case with serial PFTs following TIP diagnosis and treatment, there was only marginal improvement in DLCO at 14-week interval: 66% to 69% predicted. All patients underwent bronchoscopy, and had atypical infections such as PJP and malignant cells excluded on brochoalveolar lavage (BAL) evaluation.

## Management of TIP

Four patients received oral corticosteroids at an initial daily dose of 50mg oral prednisone or 8mg of intravenous dexamethasone within 24 hours of clinical diagnosis, followed by prolonged tapering over 4-12 weeks. The two patients who did not receive corticosteroids were offered treatment, but declined due to having a milder clinical course and concerns over developing corticosteroid-related toxicities. All patients, including the two patients who did not receive corticosteroids, received antimicrobial therapies and supportive management. All patients had the paclitaxel discontinued.

Despite the reduced DLCO on PFTs, all women recovered clinically within 6 weeks of presentation. All patients completed their subsequent adjuvant therapy, including radiotherapy, human epidermal growth factor receptor 2 (HER2) directed therapy, and endocrine therapy. For the 4 patients who required adjuvant radiotherapy, this was delayed for a median duration of 37 days (range 28-42) from the last dose of paclitaxel until the demonstration of recovery of PFTs and improvement of the radiological changes.

The three patients receiving chemotherapy in the neoadjuvant setting proceeded to uncomplicated surgery with a median time to surgery from the last dose of paclitaxel of 34 days (range 27 – 45). At surgery, pathological complete response was reported in all three patients despite an abbreviated course of systemic therapy. At 3 months after TIP diagnosis, all women remained free of respiratory symptoms, oxygen requirement, and functional impairment.

## Discussion

TIP is a relatively rare and potentially fatal toxicity in which early recognition and management is critical. Any patient receiving taxane chemotherapy who presents with a fever and/or dyspnoea should have early thoracic imaging and consideration of bronchoscopy to exclude an atypical infection, followed by the rapid initiation of steroids.

In our series, the majority of patients had evidence of an OP pattern of lung injury on CT, with bilateral ground glass changes with patchy consolidation with a predominantly sub pleural and/or peri-bronchial distribution, with the minority of patients having an NSIP pattern, consistent with published reports of paclitaxel induced pneumonitis in breast and other cancer subtypes (2, 4, 18). The point estimate of TIP incidence at our centre was 4.55%, which despite being aligned with previous TIP literature, was higher than that expected in a breast cancer specific population. This increased incidence may have been contributed to by all patients in our series receiving dose-dense doxorubicin and cyclophosphamide, prior to paclitaxel. Cyclophosphamide-induced pulmonary injury appears to be rare; the frequency is <1 percent, but is increased with concomitant use of other cytotoxic agents and can result in both early and late-onset pneumonitis (21). Additionally, doxorubicin, despite being far more commonly associated with cumulative cardiac toxicity, several reported cases of pneumonitis and, rarely, organizing pneumonia have been described (22). However, we included 132 consecutive cases of patients with breast cancer receiving paclitaxel in our series, across a year-to-year time point to reduce selection bias. Therefore, as we had a low threshold and defined clinical pathway for investigating patients with suspected TIP, the incidence described here may in fact reflect the true incidence of TIP in patients receiving paclitaxel for breast cancer. 

Predictive clinical characteristics are becoming increasingly validated for risk stratification, but besides pre-existing ILD, they rarely contraindicate treatment. Additionally, there are no currently available predictive biomarkers for TIP risk. Pre-existing ILD appears to significantly increase the incidence of TIP (14-18%), with half of these exacerbations resulting in death (23). Therefore, pre-existing ILD is should be considered to be a relative contraindication to taxane chemotherapy. Guidelines do not however recommend routine screening for baseline lung disease or to investigate asymptomatic patients for sub-clinical TIP during treatment with taxane chemotherapy.

A diagnosis of TIP is highly likely to result in compromised chemotherapy and a delay of planned subsequent curative treatment. This is especially the case with chemotherapy becoming increasingly used in the preoperative setting for triple negative and HER2 positive breast cancer subtypes. Additionally, as patients require resolution of their respiratory dysfunction in order to be fit for surgery, a dilemma that may arise regarding how long surgery can safely be delayed in the absence of systemic therapy while the breast cancer remains *in situ*. Multidisciplinary coordination between the oncologist, respiratory physician and surgeon in critical. While taxane-based chemotherapy (especially paclitaxel) increases the risk of radiation pneumonitis and radiation recall independently of prior TIP, this risk remains low (18, 21). Therefore, TIP should not contraindicate subsequent adjuvant breast radiotherapy, and when indicated and where a safe lung dose can be achieved, adjuvant radiotherapy should be completed as planned. Similarly, TIP does not contraindicate planned subsequent non-taxane based systemic therapies including HER2 or endocrine directed therapies. However, once diagnosed, we do not recommend rechallenging with taxanes. This remains a particular challenge, especially in patients with high-risk disease where treatment intensity is important in reducing risk of disease recurrence. Further investigation and validation of alternative treatment regimens and rechallenge strategies remains desirable.

Nab-paclitaxel theoretically carries a reduced risk of hypersensitivity reactions, including pneumonitis compared with conventional paclitaxel. Additionally, its use is becoming increasingly validated in the treatment of breast cancer, especially in the neoadjuvant and metastatic setting (24, 25). One case report has described successful treatment with nab-paclitaxel in a patient with NSCLC and pre-existing ILD (26). Additionally, with other rare chemotherapy toxicities such as doxorubicin-induced pancreatitis, successful rechallenge with PEGylated doxorubicin has demonstrated success (27). Further experience is needed to guide whether nab-paclitaxel may represent a legitimate candidate for rechallenge following TIP. If validated, this strategy may permit the completion of curatively intended therapy in patients experiencing TIP who are at high risk of developing metastatic disease. However, we cannot recommend this strategy without further safety data.

Finally, the rise of checkpoint inhibitor immunotherapy in the treatment of early triple-negative breast cancer adds a potential confounder. The phase 3 study KEYNOTE-522 investigated pembrolizumab versus placebo in 1174 women treated with anthracycline, cyclophosphamide, paclitaxel, and carboplatin. Paclitaxel and pembrolizumab were initiated concurrently in the study. Despite exposure to two drugs each individually associated with lung toxicity, the rate of pneumonitis of any grade was 1.3% regardless of treatment arm (28). Similarly, the phase 3 study IMpassion031 used atezolizumab versus placebo in 333 women treated with doxorubicin, cyclophosphamide, and nab-paclitaxel; the rate of any-grade pneumonitis was low at 1% in both arms (29). While there was no signal for synergistic lung toxicity in these chemo-immunotherapy combinations, the life-threatening nature of this potential side effect warrants rapid assessment of such patients presenting with respiratory decline.

## Conclusion

TIP is potentially life-threatening and affects up to one in twenty patients receiving curatively intended chemotherapy for breast cancer. Therefore, we recommend a low threshold for performing chest CT in patients receiving paclitaxel who present with dyspnoea or hypoxia. Additionally, if TIP is suspected, glucocorticoid therapy should be initiated promptly and slowly titrated with the resolution of symptoms over a period of 4-6 weeks. TIP should not contraindicate adjuvant breast radiotherapy (RT) or subsequent HER2 or endocrine directed therapies, which should proceed as planned. Finally, at present, we do not recommend rechallenging with taxane chemotherapy following TIP. However, an unanswered question remains, regarding optimal further management of patients at high-risk for developing metastatic disease. Therefore, further investigation and validation of alternative treatment regimens and rechallenge strategies remains desirable.

## Data Availability Statement

The original contributions presented in the study are included in the article/supplementary material. Further inquiries can be directed to the corresponding author.

## Ethics Statement

Written informed consent was obtained from the individual(s) for the publication of any potentially identifiable images or data included in this article.

## Author Contributions

The authors confirm contribution to the paper as follows: study conception and design: LA and EL. Data collection: LA and IW. Analysis and interpretation: LA, BL, IW, JC, LB, ES, and EL. Draft manuscript preparation: LA, BL, and EL. All authors contributed to the article and approved the submitted version.

## Conflict of Interest

The authors declare that the research was conducted in the absence of any commercial or financial relationships that could be construed as a potential conflict of interest.
